# Hydro-climatic and ecological behaviour of the drought of Amazonia in 2005

**DOI:** 10.1098/rstb.2007.0015

**Published:** 2008-02-12

**Authors:** J.A Marengo, C.A Nobre, J Tomasella, M.F Cardoso, M.D Oyama

**Affiliations:** 1 CPTEC/INPE, Rodovia Presidente Dutra12630-000 Cachoeira Paulista, São Paulo, Brazil; 2 Divisão de Ciências Atmosféricas, Centro Técnico Aeroespacial (CTA), Instituto de Aeronáutica e Espaço (IEA)Praça Marechal Eduardo Gomes, 50, 12228-904 São Jose dos Campos, São Paulo, Brazil

**Keywords:** Amazon, drought, climate change

## Abstract

In 2005, southwestern Amazonia experienced the effects of an intense drought that affected life and biodiversity. Several major tributaries as well as parts of the main river itself contained only a fraction of their normal volumes of water, and lakes were drying up. The consequences for local people, animals and the forest itself are impossible to estimate now, but they are likely to be serious. The analyses indicate that the drought was manifested as weak peak river season during autumn to winter as a consequence of a weak summertime season in southwestern Amazonia; the winter season was also accompanied by rainfall that sometimes reached 25% of the climatic value, being anomalously warm and dry and helping in the propagation of fires. Analyses of climatic and hydrological records in Amazonia suggest a broad consensus that the 2005 drought was linked not to El Niño as with most previous droughts in the Amazon, but to warming sea surface temperatures in the tropical North Atlantic Ocean.

## 1. Introduction

Drought, fire and their interactions play an important role in the carbon dynamics, vegetation–atmosphere interactions, hydrology and health of Amazon forest ecosystems, and in the livelihoods of Amazon residents. In a normal year, the region receives over 2500 mm/year rainfall. Yet, from November 2004 to the end of 2005, this region was affected by an increasingly catastrophic drought, estimated to be the worst in 40 years (Marengo *et al*. 2008).

Previous drought events occurred during El Niño years (e.g. 1926, 1983 and 1998), while the previous drought that was unrelated to El Niño was in 1964. Most Amazonian droughts during El Niño occurred in the northeastern Amazon, but the one in 2005 started in the west and southwest, and its impact spread as far as the centre and east. In 2005, from Peru to Eastern Brazil, the effects of the drought were dramatic—several major tributaries as well as parts of the main river itself contained only a fraction of their normal volumes of water, and lakes were drying up. The consequences for local people, animals and the forest itself were serious.

In a region with few roads, no river transport means no incoming supplies, and also leaves local farmers unable to sell their crops. River floodplains dried up—people could then walk and cycle in places where previously canoes and riverboats were the only means of transport. Inevitably, fishes died in millions—their bodies clogged the rivers, poisoning the water and making it impossible for local people to drink. Towns were lacking food, medicines and fuel because boats could not get through.

The causes of the drought were not related to El Niño but to (i) an anomalously warm tropical North Atlantic, (ii) a reduced intensity in northeast trade wind moisture transport into southern Amazonia during the peak summertime season, and (iii) a weakened upward motion over this section of Amazonia, resulting in reduced convective development and rainfall. The drought conditions were intensified during the dry season until September 2005 when humidity was lower than normal and air temperatures 3–5°C warmer than normal. At this time, the river levels were well below normal and navigation was not possible in many parts of the Solimões River. Rains returned in October 2005 and generated flooding after February 2006 (Marengo *et al*. 2008).

To make matters worse, as the rainforest became increasingly dry, damaging wildfires regularly broke out across the region, destroying thousands of hectares of trees. Owing to the extended dry season in the region, forest fires affected a part of southwestern Amazonia. The fires occurred mainly where there was human activity, which could ignite them. In the Brazilian State of Acre, in southwestern Amazonia, CPTEC/INPE (www.cptec.inpe.br/queimadas) has reported that the number of fire pixels detected using the NOAA12 satellite tripled to nearly 2800 at its peak in September 2005, compared with 800 in 2004. In Amazonas, the number of fire pixels in September 2004 was 760 while in September 2005 it nearly tripled to 2166. Amazonian deforestation and fires account for more than 75% of Brazil's greenhouse gas emissions and place it among the top four contributors to global climate change.

Reviews on the spatial extent of the droughts and fire response to the 2005 drought are found in Brown *et al*. (2006) and Aragão *et al*. (2007). They suggest that the 2005 drought was characterized by the intensification of the dry season in southwestern Amazonia, favouring conditions for the propagation of fires; at the time the levels of many rivers in the region were below normal. During 2005, the annual cumulative number of fires in Amazonia increased 33% in relation to the 1999–2005 mean. In the State of Acre, at the centre of the 2005 drought, the area of leakage forest fires was more than five times greater than the area directly deforested. Fire leakage into flammable forests may be, therefore, the major agent of biome transformation in a scenario of increased drought frequency in this region.

The present study focuses on the hydro-climatic characteristics of the 2005 drought in Amazonia extending on the observational analyses from Marengo *et al*. (2008) and the ecological studies by Aragão *et al*. (2007). The present study is directed to (i) provide a new detailed hydrological analysis of the drought and (ii) assess the near-surface climatic conditions that led to the propagation of fires during this drought.

## 2. Data and methodology

For tropical South America, data from the Global Precipitation Climatology Center (GPCC; Rudolf *et al*. 1994; Rudolf & Schneider 2005) were used. The GPCC gauge-based gridded precipitation dataset is available for the global land surface only. The quality control is carried out with respect to outliers and homogeneity (both test and removal) as well as the interpolation, and gridding is carried out as thoroughly as possible in order to obtain optimal results (Rudolf *et al*. 1994; Beck *et al*. 2005). The GPCC datasets are available on a 1.0° horizontal resolution. No comparisons were made for previous drought events (1963–1964, 1982–1983) since the GPCC monitoring product has only been available since 1996. These data are available as mean monthly precipitation totals and anomalies from the 1961–1990 long-term mean, and we focus on the seasonal rainfall anomalies during 2005 and 2006.

River discharge and levels datasets from gauging sites in the Brazilian Amazonia were provided by Agencia Nacional de Aguas ANA (National Water Authority) and the administration of the Port of Manaus. Most of the river data (levels and streamflow) are available from the 1930s, with the exception of the levels of the Negro River at Manaus Port that are available from 1903. The data of the Solimões River at Fonte Boa and the Rio Branco river at Rio Branco are available from 1931. River information from the Amazon River records at Óbidos is available from 1968. For the purposes of this study, we used the common record between 1970 and 2006. Near-surface relative humidity (%; RH), as well as sensible and latent heat fluxes were derived from the 850 hPa level fields of the NCEP/NCAR global reanalyses (Kalnay *et al*. 1996), which are on a 2.5° horizontal resolution.

Fire data are from the fire monitoring programme at CPTEC/INPE, based on active-fire detections using the NOAA12 satellite (Setzer & Malingreau 1996). These data have been a major source of information on fire activity for ecological and atmospheric research in Amazonia, and are provided daily at the spatial resolution of 1 km at Nadir. To avoid false positives due to solar reflection, we used data from afternoon overpasses covering the study region at approximately 20.00 GMT. Here, the detections were aggregated at monthly time scale and filtered out for locations outside the Brazilian Amazonia.

## 3. Results and discussions

### (a) Climatic features of the drought of 2005

**Figure 1:**
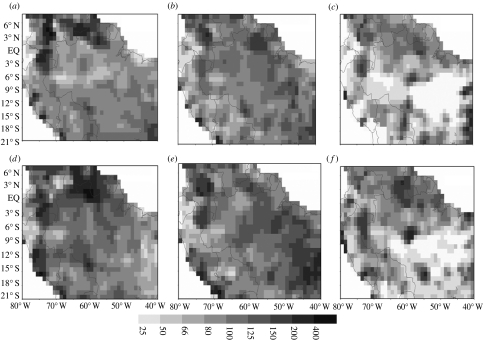
Seasonal rainfall anomaly maps for tropical South America from December 2004–February 2005 to June–August 2006. (*a*) December 2004–February 2005, (*b*) March–May 2005, (*c*) June–August 2005, (*d*) December 2005–February 2006, (*e*) March–May 2006 and (*f*) June–August 2006. Values as shown as percentages of the 1961–1990 long-term mean. Data are from GPCC-Monitoring product available at 1.0° latitude/longitude gridbox area. Grey tones indicate percentages (%) from the mean.

The rainfall records indicate that the basins in the southern and western Amazon regions were the most affected by the drought during 2005, especially during the peak of the rainy season in early austral summer. Figure 1 shows a large reduction in rainfall during November 2004 to January 2005 and then after April 2005, and this variability was reflected in the river levels in the major Amazon River tributaries such as the Solimões River starting in May 2005. The dry season, June to August 2005, was more intense than normal in western Amazonia, with rainfall that sometimes reached 25% of the normal value during the season in southern Amazonia.

Different from the intense droughts during El Niño years 1983 and 1998, the drought in 2005 was concentrated in western and southern Amazonia, and not so much in northern or eastern Amazonia. As in 1998, the 2005 drought was also characterized by extended fires in the region, suggesting that the drought–fire interaction is not necessarily restricted to El Niño or El Niño-like events. In fact, the relation would also involve the length of the dry season, the intensity of the rainy season and the regional water balance, where high air temperatures and reduced atmospheric moisture and intense evaporation may affect the soil moisture content in the presence of a below-normal rainy season.

### (b) Hydrological features of the drought

**Figure 2:**
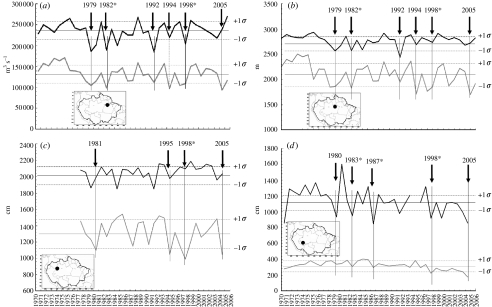
River level/streamflow series at different gauge stations for rivers with basins extending in central and eastern Amazonia, and in southern and western Amazonia in Brazil, Bolivia and Peru during 1970–2006, for the high/low season. (*a*) Amazon River at Óbidos, for May to June (solid black line)/September to October (solid grey line; in m^3^ s^−1^); (*b*) Negro River at Manaus, for June to July (solid black line)/September to October (solid grey line; in m); (*c*) Solimões River at Fonte Boa for May to June (solid black line)/September to October (solid grey line) and (*d*) Rio Branco River at Rio Branco, for February to March (solid black line)/August to September (solid grey line; both in cm). Arrows show year with drought. Mean and s.d. are shown in each panel for the season of high and low river stands. Asterisk indicates El Niño year.

The time series of monthly levels/discharges of the Amazon River and three of its main tributaries, Negro, Solimões and Branco Rivers (the latter two have their drainage area in southern Amazonia), are shown in figure 2*a*–*d*, together with the season of high and low river stands for each gauge station.

In terms of the long-term means, the Branco River (Southwest Amazonia) reaches its peak during February, while the minimum usually is recorded in September. The Amazon River at the Óbidos gauge site, on average, shows the season of maximum flow during May to June. After this peak, there is a gradual recession of the minimum in November. Therefore, the lag time between the peak at the Branco River and the Amazonas River at Óbidos is, on average, four months, while the lag time between lows is only two months. The reason for these differences in the lag time is related to the hydrological regime of the western and northern parts of the basin: the Solimões River at Tabatinga (on the Brazilian–Colombian border) for instance, peaks around May (one month before the peak of Óbidos), and has a minimum around September (two months before the peak at Óbidos). Consequently, the maximum discharge at Óbidos is a combination of several rivers that show a different annual cycle on their flows, and these contributions reflect on the timing of the Amazon river discharges at Óbidos. The minimum, however, is reached simultaneously in most tributaries during September, and the signal arrives simultaneously in Óbidos during November.

Some of the river series show lower values during the El Niño events between 1982 and 1983 and to a lesser degree in 1998. The water levels were very low during the drought of 2005, and in some cases the values were lower than 1 s.d., which is particularly significant since they occurred during the season when the levels were minimum.

The discharges of the Amazon River at Óbidos (figure 2*a*) show values during the low season, September to October 2005, below 100 000 m^3^ s^−1^, which are the lowest since 1970, while the values in the high season, May to June, were slightly above normal. In other drought years such as 1979, 1982, 1994 and 1998, the reduction in discharge is detected in both the high and low seasons. The Negro River was approximately 20 cm above the normal during the June to July high season in 2005, and from January to July 2005 the levels were approximately 1–2 m above normal in Manaus (figure 2*b*). Since August 2005, the river levels dropped to values approximately 3 m below normal, and during the low season September to October the values were almost 2.5 m below normal. It reached 18.61 m in September 2005 (September average=22.30 m). For comparison, the Rio Negro level in Manaus reached 21.74 m in September 2004, and it dropped almost 4 m below normal by September 2005.

The water level of the Negro River in Manaus is a combination of the signal produced by the Rio Negro itself and the nearby Solimões River. Since the discharge of the Solimões River is, on average, three times greater than the Negro River, the water levels of the Negro River in Manaus are strongly influenced by the backwater effect produced by the Solimões at the confluence of both the rivers. The levels of the Rio Solimões also experience large drops during the September to October season in 1995 and 1998, larger in magnitude than those of 2005 (figure 2*c*). A large drop in river levels in Manaus is observed during the September to October low season while during the June to July high season the levels were near normal. The drop in the levels at the Manaus gauge site during the September to October low season was due to the drop in the levels in the same season of the Solimões River upstream of the Manaus site.

The values of the Branco River (figure 2*d*) also show low levels in 1998 and in previous El Niño years, but the lowest of the record was in 2005. These levels, both during the February to March high and during the August to September low seasons, experience a negative trend since the beginning of the 1970s.

It is clear that the drought of 2005 has characteristics different from the previous recorded events, since it strongly affected the southwest portion of the basin. Owing to the sheer size and significant travel time of the Amazon Basin, the contribution of that part of the basin occurred when the water levels were already receding at the Manaus gauge site. Therefore, rainfall anomalies strongly affected the water level at Manaus by the time of year when the stages are normally minimum, increasing the recession in October 2005, while the peak discharge remained unaffected. In all gauge sites, for the four rivers considered, the low season values in 2005 were more than 1 s.d. lower than the mean, something that was observed to some degree in 1998, but in that year the drops were also observed in the high season.

Since the drought of 2005 affected western and southern Amazonia, it is clear that several tributaries that drain extensive areas of the southern part of the Amazon Basin were affected. It is important to note that the largest contribution areas of the Amazon River are located to the south of the basin. Therefore, lower than normal contribution from the southern part of the basin affected the Amazon River discharges along the main river, which explains why the drought impacts increased downstream.

Even though the records are relatively short, a small negative trend in low level/discharge season has been detected since the beginning of the 1970s, most of the river time series shown in figure 2. The same tendency was observed in the Amazon River discharge series at Iquitos (SENAMHI-National Meteorological Service from Peru). The contribution from Solimões and Madeiras Rivers is approximately 49 and 16%, respectively, of the Amazon River discharge (Molinier *et al*. 1996), therefore slightly negative discharges of the Amazon River measures at Óbidos might be explained by the negative trends in both the Solimões and the Madeiras Rivers.

### (c) Near-surface climate conditions and fire risk during the drought of 2005

The onset and propagation of fires depend on the soil moisture content, which is related to the length of the dry season, and the hydrological signal, which depends on the quality of the rainy season. Hence, it is possible to have a year that, from the hydrological point of view, may be normal with plenty of rain during the rainy season, but if the dry season is longer than normal the year can be considered as dry from the ecological point of view. On the other hand, organic matter and litter accumulate continuously, and during normal or wet years fire is inhibited to propagate because the litter and organic matter are not sufficiently dry and they accumulate from one year to another. When a drought impacts Amazonia, the litter and organic material dry up and can become ‘fuel’ for a fire, and with an ignition (natural or human induced) an intense fire season can develop. In 2004 and 2005, the dry seasons were very rigorous in almost all Amazonia, the rainy season was deficient only in southwest Amazonia, and the fires were more intense and frequent in that section of Amazonia.

**Figure 3:**
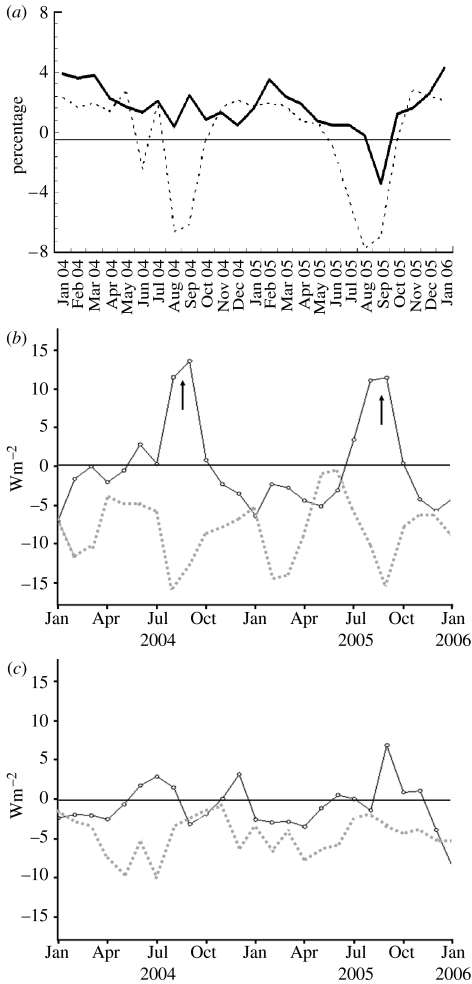
(*a*) Time series of 850 hPa NCEP reanalyses RH anomalies (%) for northern Amazonia (solid line) and southern Amazonia (dashed line) from January 2004 to January 2006; (*b*) time series of 850 hPa NCEP reanalyses latent (dashed line) and sensible (solid line) heat (W m^−2^) for southern Amazonia from January 2004 to January 2006; (*c*) as in (*b*) but for northern Amazonia. Anomalies are in relation to the 1968–2006 climatology: (*a*) mean of 92% and s.d. of 1.5% for northern Amazonia, and mean of 85% and s.d. of 2.5% for southern Amazonia; (*b*) for northern Amazonia, mean of 136 W m^−2^ and s.d. of 5.4 W m^−2^ for latent heat, and mean of 10.4 W m^−2^ and s.d. of 2.7 W m^−2^ for sensible heat; (*c*) for southern Amazonia, mean of 117 W m^−2^ and s.d. of 6.6 W m^−2^ for latent heat, and mean of 23.5 W m^−2^ and s.d. of 5.7 W m^−2^ for sensible heat.

Figure 3*a*–*c* shows the time series of RH anomalies in 850 hPa, and latent and sensible anomalies for northern and southern Amazonia (regions defined in Marengo *et al*. (2008) according to the different rainfall annual cycle). These fields were derived from the NCEP reanalysis from January 2004 to January 2006, and compare quite well with the surface meteorological observations in the region. In southern Amazonia, starting in June 2005, the RH anomalies reached up to 8% below normal, indicating conditions drier than normal and favouring the drying up of the dead biomass. According to CPTEC reports, maximum air temperatures were 4–5°C above normal during June to September 2005 in southern Amazonia. These conditions, RH decrease and temperature increase, were consistent with the marked negative (positive) latent (sensible) heat anomalies indicated by solid/dashed (black) lines in figure 3*b*,*c*. On the other hand, both conditions, decrease in low-level atmospheric moisture (associated to less latent heat) and increase in surface temperature (associated to more sensible heat), mean water stress and would favour the occurrence and propagation of fire, particularly during the wintertime dry season. In northern Amazonia, the RH was closer to normal in 2004 and most of 2005, while during the dry season of 2005 it was approximately 4% below normal. Latent and sensible heat anomalies did not show marked anomalies. Therefore, the situation certainly was less dramatic for northern than for southern Amazonia.

**Figure 4:**
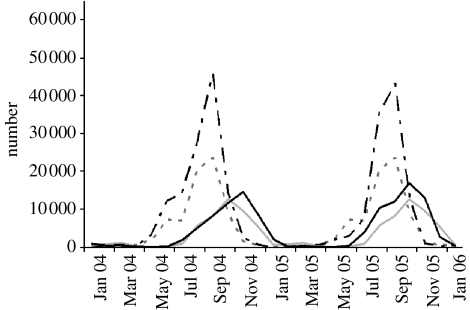
Time series of the number of fire pixels detected in the Brazilian Amazonia from January 2000 to January 2006. The monthly number of fire pixels from January 2004 to January 2006 is shown as a solid black line for the north (between 6 and 7° S) and as a dashed black line for the south (between 7 and 18° S). The average number of fire pixels during 2000–2003 is shown as solid grey line for the north and as dashed grey line for the south. Fire detections are from CPTEC/INPE based on NOAA12 afternoon overpasses approximately 20 GMT.

Figure 4 shows the time series of the number of fire pixels detected with NOAA 12 in the north and the south of the Brazilian Amazonia, from January 2000 to January 2006. The monthly number of fire pixels detected during this period is represented by the solid black line for the north of the region and the dashed black line for the south. To provide a reference for these values in relation to previous years, the grey lines display the average number of fire pixels during 2000–2003 for each region and specific month. The solid grey line displays monthly values for the north and the dashed grey line for the south. As shown, fire detection in both the subregions reflects the seasonality in precipitation.

While fire pixels were generally detected in the dry season as expected, there is an indication of overall increase in fire activity in 2004 and 2005. In both subregions, the number of fire pixels was higher during most of this period (black lines) than in the four previous years (grey lines). The results also present different patterns of changes between the two subregions. First, the results indicate that the increase in fire activity was more accentuated in the south of the region. As shown, for most of 2004 and 2005 the relative differences in the number of fire pixels between dashed lines (south) are higher than those between solid lines (north). Second, the relative increase in the fire activity for 2004 in the north is noticeable only from November. Before that, the solid grey and black lines are very similar.

## 4. Discussions and conclusions

From the observational point of view, it is concluded that the drought that affected southern and western Amazon during summer 2005 was due to the reductions in rainfall from December 2004 to February 2005. The main characteristic of the drought was the lower river levels and discharges during the May to July peak season, a consequence of the reduction in rainfall a few months before. This season of low river stands was also accompanied by rainfall amounts over southern Amazonia that were approximately 25–40% of the norm, accompanied by a drier and warmer atmosphere that helped in the set-up and propagation of forest fires in the states in southern and western Amazonia. This drought particularly impacted the onset and peak of the rainy season during spring and early summer of 2005 and, to a lesser degree, during autumn in northern and central Amazonia.

The drought also favoured the occurrence of fires. The main patterns in our fire analysis reflect the major climate features in 2004 and 2005. Generally, both the occurrence and the overall increase in fire activity reflected the timing and the negative anomalies of the wetness conditions. As shown, the majority of fires were detected in the dry season and the number of detections in the period was higher than in previous years (figure 4), consistent with the occurrence of the drought (figure 3). In addition, the differences in intensity of the fire-activity change between north and south of Amazonia can also be related to the features of the drought in these subregions. In the north, the increase in fire activity was relatively smaller than that in the south, and occurred mostly in the dry season of 2005, similar to the occurrence of negative anomalies of humidity in that region (figure 3*a*). In the south, the relative increase in fire detections was more intense than in the north and occurred during most of the dry seasons in 2004 and 2005. In that region, negative anomalies in RH were also more intense than in the north and happened in both the years (figure 3*a*).

In the northern-central part of the Amazon region in the basin of the Rio Negro, the sudden drop of the levels at Manaus since July 2005 was not due to reduced rainfall in the northwestern Amazonia but due to the effect of the low flow of Solimões River into the measurements at Manaus due to reduced rainfall in its basin. This was also detected in the discharges of the Amazon River at Óbidos.

Therefore, the anomalously low levels in many of the rivers in southwestern Amazonia during the autumn to wintertime peak season was due to the reductions in rainfall in that region during the summertime peak season three to four months before. The wintertime season was also accompanied by large reductions in rainfall and a dry and warm atmosphere that favoured fire ignition and propagation. The levels in Manaus were low only during July but due to the effect of the lower levels of the Solimões that somewhat affected the levels at Manaus. This drought was different from those associated with El Niño, where anomalies impact both summer and autumn rainfall in central and eastern Amazonia, producing very large drops in the Manaus levels, as in 1926, 1983 and 1998. The drought of 2005 was somewhat similar to that between 1963 and 1964.

## References

[bib1] Aragão L.E.O.C, Mahli Y, Roman-Cuestas R.M, Saatchi S, Anderson L, Shimabukuro Y (2007). Fingerprints of the 1997/1998 and 2005 droughts in Amazonian rainforests. Geophys. Res. Lett.

[bib2] Beck, C., Grieser, J. & Rudolf, B. 2005 *A new monthly precipitation climatology for the global land areas for the period 1951 to 2000* DWD, Klimastatusbericht 2004.

[bib3] Brown I.F, Schroeder W, Setzer A, Maldonado M, Pantoja N, Duarte A, Marengo J (2006). Fires in rain forests of southwestern Amazonia: multi-national satellite imagery for monitoring and for informing the public. EOS Trans.

[bib5] Kalnay E (1996). The NCEP/NCAR 40 year reanalysis project. Bull. Am. Met. Soc.

[bib6] Marengo J.A, Nobre C.A, Tomasella J, Oyama M.D, de Oliveira G.S, de Oliveira R, Camargo H, Alves L.M, Brown I.F (2008). The drought of Amazonia in 2005. J. Clim.

[bib7] Molinier M, Guyot J.L, de Oliveira E, Guimarães V (1996). Les régimes hydrologiques de l'Amazone et de ses affluents. L'hydrologie tropicale: géoscience et outil pour le développement, Paris, Mai 1995. IAHS Series of Proceedings and Reports.

[bib8] Rudolf, B. & Schneider, U. 2005 Calculation of gridded precipitation data for the global land-surface using *in-situ* gauge observations. In *Proc. 2nd workshop of the Int. Precipitation Working Group IPWG, Monterey, October 2004*

[bib9] Rudolf B, Hauschild H, Rueth W, Schneider U, Desbois M, Desalmand F (1994). Terrestrial precipitation analysis operational method and required density of point measurements. NATO ASI I/26, global precipitations and climate change.

[bib10] Setzer A, Malingreau J.P, Levine J.S (1996). AVHRR monitoring of vegetation fires in the tropics: towards a global product. Biomass burning and global change.

